# Supplementary material for: The Evolution and Characterization of the RNA Interference Pathways in Lophotrochozoa
Source: Genome Biol Evol. 2024 May 7;16(5):evae098. doi: 10.1093/gbe/evae098 (PMC11114477; doi:10.1093/gbe/evae098)
Supplement: evae098_Supplementary_Data [file evae098_supplementary_data.zip › Supplementary_materials.zip!/selected_miRNAs/NoGe_selected_miRNAs/mirdeep_pdf_files/NMRB01000152.1_Notospermus_geniculatus_strain_Ushimado_scaffold152,_whole_genome_shotgun_sequence_5844.pdf]

Provisional ID : NMRB01000152.1_Notospermus_geniculatus_strain_Ushimado_scaffold152,_whole_genome_shotgun_sequence_5844
Score total : 92455
Score for star read(s) : 3.9
Score for read counts : 92446.2
Score for mfe : 1.9
Score for randfold : 0
Score for cons. seed : 3
Total read count : 181341
Mature read count : 181247
Loop read count : 0
Star read count : 94

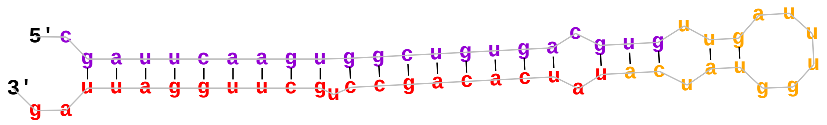

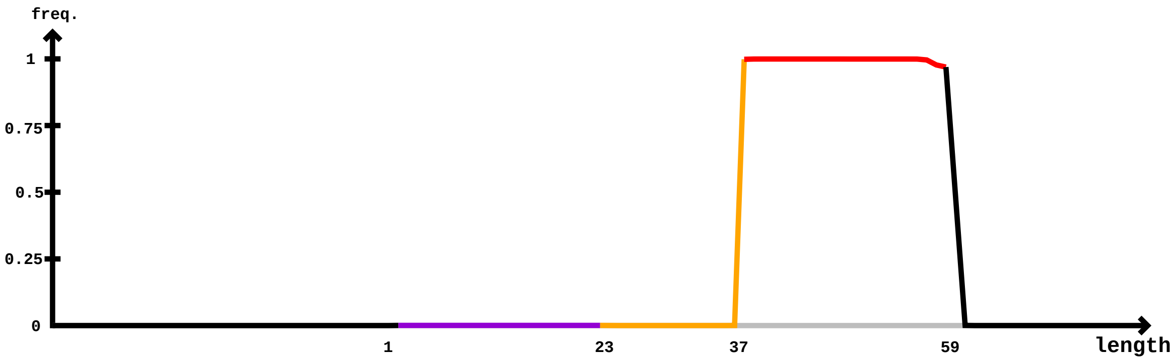


```
                                       Star                                  Mature
5'- gagaaguguacaaugaaaguuuggcaucugccuucgauucaaguggcugugacguguugauuugguaucauaucacagccugcuuggauuagagguugguugucaguugugc   -3'   obs
    gagaaguguacaaugaaaguuuggcaucugccuucgauucaaguggcugugacguguugauuugguaucauaucacagccugcuuggauuagagguugguugucaguugugc         exp
    .......(((((((.......(((((((.(((((.(((((((((((((((((.(((.((......)).))).)))))))).)))))))))).))))).)).))))))))))))   reads   mm   sample
    ............................................................uuugguaucauaucacagccu...............................   4       0    Fm1
    .....................................................................Cuaucacagccugcuuggauuag....................   2       1    Fm1
    .....................................................................auaucacagccugcuuggauuag....................   3       0    Fm1
    ......................................................................uaucacagccugcuugga........................   4       0    Fm1
    ......................................................................uaucacagccugcuuggau.......................   6       0    Fm1
    ......................................................................uaucacagcUugcuuggau.......................   3       1    Fm1
    ......................................................................uaucacagccugcuugUauu......................   1       1    Fm1
    ......................................................................uaucacagccugcuuUgauu......................   7       1    Fm1
    ......................................................................uaucacagccugcuuggauu......................   127     0    Fm1
    ......................................................................uaucacagcAugcuuggauu......................   1       1    Fm1
    ......................................................................uaucacagccugcuugAauu......................   1       1    Fm1
    ......................................................................uaucacagccCgcuuggauu......................   1       1    Fm1
    ......................................................................uaucacagcUugcuuggauu......................   5       1    Fm1
    ......................................................................uaucacagccugcuugAauua.....................   1       1    Fm1
    ......................................................................uaucacagccugcuuggauuU.....................   6       1    Fm1
    ......................................................................uaucacagccugcuugCauua.....................   1       1    Fm1
    ......................................................................uaucacagccugcuuggauua.....................   57      0    Fm1
    ......................................................................uaucacagcUugcuuggauua.....................   2       1    Fm1
    ......................................................................uGucacagccugcuuggauuag....................   1       1    Fm1
    ......................................................................uaucUcagccugcuuggauuag....................   1       1    Fm1
    ......................................................................uaucGcagccugcuuggauuag....................   3       1    Fm1
    ......................................................................uaucaGagccugcuuggauuag....................   1       1    Fm1
    ......................................................................uaCcacagccugcuuggauuag....................   1       1    Fm1
    ......................................................................uaGcacagccugcuuggauuag....................   1       1    Fm1
    ......................................................................uaAcacagccugcuuggauuag....................   1       1    Fm1
    ......................................................................Naucacagccugcuuggauuag....................   1       1    Fm1
    ......................................................................uaucacagccugcuuggUuuag....................   22      1    Fm1
    ......................................................................Caucacagccugcuuggauuag....................   1       1    Fm1
    ......................................................................uaucacagccugcuuggauuag....................   4460    0    Fm1
    ......................................................................uUucacagccugcuuggauuag....................   1       1    Fm1
    ......................................................................uaucacagcUugcuuggauuag....................   31      1    Fm1
    ......................................................................uaucacGgccugcuuggauuag....................   14      1    Fm1
    ......................................................................uaucCcagccugcuuggauuag....................   1       1    Fm1
```

```
                                  Star                          Mature
gagaaguguacaaugaaaguuuggcaucugccuucgauucaaguggcugugacguguugauuugguaucauaucacagccugcuuggauuagagguugguugucaguugugc
......................................................................uaucacagcGugcuuggauuag....................  1    1  Fm1
......................................................................uaucacagccugcuuUgauuag....................  3    1  Fm1
......................................................................uaucacagccGgcuuggauuag....................  10   1  Fm1
......................................................................uaucacagccCgcuuggauuag....................  2    1  Fm1
......................................................................uaucacagccugcuuggaGuag....................  2    1  Fm1
......................................................................uaucacagccugcuugUauuag....................  5    1  Fm1
......................................................................uaucacagccugcuuggCuuag....................  23   1  Fm1
......................................................................uaucacaAccugcuuggauuag....................  1    1  Fm1
......................................................................uaucacagccugcuuggauuaA....................  1    1  Fm1
......................................................................uaucacagUcugcuuggauuag....................  44   1  Fm1
......................................................................uauUacagccugcuuggauuag....................  1    1  Fm1
......................................................................uaucacagccugcuuggauuUg....................  2    1  Fm1
......................................................................uaucacagccAgcuuggauuag....................  9    1  Fm1
......................................................................uaucacagccugcuuggauuGg....................  6    1  Fm1
......................................................................uaucacUgccugcuuggauuag....................  1    1  Fm1
......................................................................uaucacagccugcuugAauuag....................  7    1  Fm1
......................................................................uaucacagccugcuuggGuuag....................  16   1  Fm1
......................................................................uaucacagccugcuGggauuag....................  1    1  Fm1
......................................................................uaucaUagccugcuuggauuag....................  2    1  Fm1
......................................................................uaucacagccugcuugCauuag....................  11   1  Fm1
......................................................................uaucacagccugcuuggauuaU....................  8    1  Fm1
......................................................................uaucacagccugcuuggauuaga...................  791  0  Fm1
......................................................................uaucacagccugcuuggauuagU...................  242  1  Fm1
......................................................................uaucacagccAgcuuggauuaga...................  4    1  Fm1
......................................................................uaucacagccugcuuggGuuaga...................  2    1  Fm1
......................................................................uaucacagccugcuuggauuagG...................  7    1  Fm1
......................................................................uaucacagccuUcuuggauuaga...................  1    1  Fm1
......................................................................uaucacagccugcuugCauuaga...................  4    1  Fm1
......................................................................uaucacagUcugcuuggauuaga...................  1    1  Fm1
......................................................................uaucacagccugcuuggCuuaga...................  7    1  Fm1
......................................................................uaucacagccugcuuggUuuaga...................  2    1  Fm1
......................................................................uaucacagccugUuuggauuaga...................  4    1  Fm1
......................................................................uaucacagccugcuugAauuaga...................  1    1  Fm1
......................................................................uaucacagccGgcuuggauuaga...................  1    1  Fm1
......................................................................uaucacGgccugcuuggauuaga...................  7    1  Fm1
......................................................................uaucacagcUugcuuggauuaga...................  5    1  Fm1
......................................................................uaucacagccugcuugUauuaga...................  1    1  Fm1
......................................................................uaucacagccugcuuggauuagaU..................  1    1  Fm1
.......................................................................aucacagccugcuuggauuag....................  1    0  Fm1
.......................................................................aucacagccugcuuggauuaga...................  2    0  Fm1

..................................cgauucaaguggcugugacgug........................................................  90   0  Fm2
..................................cgauucaaguAgcugugacgug........................................................  1    1  Fm2
..................................cgauucaaguggcuguCacgug........................................................  1    1  Fm2
........................................................uugauuugguaucauaucacagccu...............................  13   0  Fm2
.....................................................................auaucacagccugcuuggauu......................  4    0  Fm2
.....................................................................auaucacagccugcuuggauuag....................  10   0  Fm2
......................................................................uaucacagccugcAuggau.......................  1    1  Fm2
......................................................................uaucacagccugcuuggau.......................  25   0  Fm2
......................................................................uaucacagccugcuuggauG......................  1    1  Fm2
......................................................................uaucacagccugcuuggUuu......................  2    1  Fm2
......................................................................uaucacagccugcuuUgauu......................  2    1  Fm2
......................................................................uaucacagccugcuuggCuu......................  1    1  Fm2
......................................................................uaucacagUcugcuuggauu......................  7    1  Fm2
......................................................................uaucacagccCgcuuggauu......................  1    1  Fm2
......................................................................uaucacagccAgcuuggauu......................  1    1  Fm2
......................................................................uaucacagccugcuuggauu......................  210  0  Fm2
......................................................................uaucacagccugUuuggauua.....................  1    1  Fm2
......................................................................uaucCcagccugcuuggauua.....................  1    1  Fm2
......................................................................uaucacagccugcuuAgauua.....................  1    1  Fm2
......................................................................uaucacagccugcuuggauuU.....................  17   1  Fm2
......................................................................uaucacagccugcuuggauua.....................  134  0  Fm2
......................................................................uaucacagccugcuuggauuG.....................  6    1  Fm2
......................................................................uUucacagccugcuuggauuag....................  1    1  Fm2
......................................................................uaucacagccugcuugAauuag....................  28   1  Fm2
......................................................................uaucGcagccugcuuggauuag....................  8    1  Fm2
......................................................................uaucacagccugcGuggauuag....................  2    1  Fm2
......................................................................uaucacagccugcuuggauuUg....................  12   1  Fm2
......................................................................uaucacagccuAcuuggauuag....................  4    1  Fm2
......................................................................uaucaAagccugcuuggauuag....................  2    1  Fm2
```

Star Mature

```
gagaaguguacaaugaaaguuuggcaucugccuucgauucaaguggcugugacguguugauuugguaucauaucacagccugcuuggauuagagguugguugucaguugugc
......................................................................uaucacagccugcuuggauCag....................   1       1   Fm2
......................................................................uaucacGgccugcuuggauuag....................   65      1   Fm2
......................................................................uCucacagccugcuuggauuag....................   1       1   Fm2
......................................................................uaucaGagccugcuuggauuag....................   1       1   Fm2
......................................................................uaucacaUccugcuuggauuag....................   7       1   Fm2
......................................................................uaucacagccugcuCggauuag....................   2       1   Fm2
......................................................................uaucacagccugcuugCauuag....................   56      1   Fm2
......................................................................uaucacagccugcAuggauuag....................   2       1   Fm2
......................................................................uaGcacagccugcuuggauuag....................   6       1   Fm2
......................................................................Caucacagccugcuuggauuag....................   4       1   Fm2
......................................................................Gaucacagccugcuuggauuag....................   1       1   Fm2
......................................................................uaucacagccugUuuggauuag....................   22      1   Fm2
......................................................................uaucacNgccugcuuggauuag....................   1       1   Fm2
......................................................................uaucacUgccugcuuggauuag....................   2       1   Fm2
......................................................................uaucacagccCgcuuggauuag....................   15      1   Fm2
......................................................................uaucacagccugcuuggauuGg....................   11      1   Fm2
......................................................................uaucUcagccugcuuggauuag....................   3       1   Fm2
......................................................................uaucacagUcugcuuggauuag....................   94      1   Fm2
......................................................................uaucacagccugcuuggauuaU....................   27      1   Fm2
......................................................................uaucacaAccugcuuggauuag....................   1       1   Fm2
......................................................................uaucacagccugcuuggaAuag....................   3       1   Fm2
......................................................................uaucacagccGgcuuggauuag....................   18      1   Fm2
......................................................................uaucacagccugcuuggauuag....................   18305   0   Fm2
......................................................................uauUacagccugcuuggauuag....................   2       1   Fm2
......................................................................uaucacagccAgcuuggauuag....................   46      1   Fm2
......................................................................uaucacagccugcuuggaGuag....................   1       1   Fm2
......................................................................uaucacagccugcuuggGuuag....................   63      1   Fm2
......................................................................uauAacagccugcuuggauuag....................   4       1   Fm2
......................................................................uaucacagccugcuuggUuuag....................   57      1   Fm2
......................................................................uaCcacagccugcuuggauuag....................   3       1   Fm2
......................................................................uaucacagccugcuuUgauuag....................   6       1   Fm2
......................................................................uaAcacagccugcuuggauuag....................   6       1   Fm2
......................................................................uaucacagccuUcuuggauuag....................   2       1   Fm2
......................................................................uaucacagccugcuugUauuag....................   32      1   Fm2
......................................................................uaucaUagccugcuuggauuag....................   7       1   Fm2
......................................................................uGucacagccugcuuggauuag....................   5       1   Fm2
......................................................................uaucaNagccugcuuggauuag....................   5       1   Fm2
......................................................................uaucacagccugcuAggauuag....................   2       1   Fm2
......................................................................Naucacagccugcuuggauuag....................   14      1   Fm2
......................................................................uaucacagccugcuuCgauuag....................   1       1   Fm2
......................................................................uaucacagccugcuuggaCuag....................   2       1   Fm2
......................................................................uaucacagAcugcuuggauuag....................   1       1   Fm2
......................................................................uaucacagccugcuuggauuaA....................   11      1   Fm2
......................................................................uaucacagccugcuuggauuCg....................   1       1   Fm2
......................................................................uaucacagccuCcuuggauuag....................   2       1   Fm2
......................................................................uaucacagcAugcuuggauuag....................   2       1   Fm2
......................................................................uaucacagccugcCuggauuag....................   1       1   Fm2
......................................................................uaucCcagccugcuuggauuag....................   2       1   Fm2
......................................................................uaucacagccugcuuggCuuag....................   82      1   Fm2
......................................................................uaucacagcUugcuuggauuag....................   23      1   Fm2
......................................................................uaucacagccCgcuuggauuaga...................   1       1   Fm2
......................................................................uaucacaUccugcuuggauuaga...................   1       1   Fm2
......................................................................uaCcacagccugcuuggauuaga...................   1       1   Fm2
......................................................................uaucacagccugcuuggauuagG...................   36      1   Fm2
......................................................................uaucacagccGgcuuggauuaga...................   4       1   Fm2
......................................................................uaucaAagccugcuuggauuaga...................   1       1   Fm2
......................................................................uaucacGgccugcuuggauuaga...................   10      1   Fm2
......................................................................uaucacagccugcuCggauuaga...................   2       1   Fm2
......................................................................uGucacagccugcuuggauuaga...................   1       1   Fm2
......................................................................uaucacaCccugcuuggauuaga...................   1       1   Fm2
......................................................................uaucacagccugcuugCauuaga...................   9       1   Fm2
......................................................................uaucacagccugAuuggauuaga...................   1       1   Fm2
......................................................................uaucacagccugcuuAgauuaga...................   1       1   Fm2
......................................................................uaucacagccugcuugUauuaga...................   2       1   Fm2
......................................................................uaucacagccugcuAggauuaga...................   1       1   Fm2
......................................................................uaucUcagccugcuuggauuaga...................   1       1   Fm2
......................................................................uaucacagccugcuuggauuaga...................   2820    0   Fm2
......................................................................uaucacagccugcuuggauuagU...................   872     1   Fm2
......................................................................uaucacagcUugcuuggauuaga...................   1       1   Fm2
......................................................................uaucacagccugUuuggauuaga...................   9       1   Fm2
```

```
                                  Star                              Mature
gagaaguguacaaugaaaguuuggcaucugccuucgauucaaguggcugugacguguugauuugguaucauaucacagccugcuuggauuagagguugguugucaguugugc
......................................................................uaucacagccugcuuggCuuaga...................   12      1     Fm2
......................................................................uaucacCgccugcuuggauuaga...................   2       1     Fm2
......................................................................uaucGcagccugcuuggauuaga...................   1       1     Fm2
......................................................................uaucacagccugcuuggauGaga...................   1       1     Fm2
......................................................................uaucacagUcugcuuggauuaga...................   14      1     Fm2
......................................................................uaucacagccAgcuuggauuaga...................   7       1     Fm2
......................................................................uaucacagccugcuugAauuaga...................   5       1     Fm2
......................................................................uUucacagccugcuuggauuaga...................   1       1     Fm2
......................................................................uaucacagccugcuuggGuuaga...................   8       1     Fm2
......................................................................uaucacagccugcuuggauuagC...................   1       1     Fm2
......................................................................uaucacagccugcuuggUuuaga...................   12      1     Fm2
......................................................................uaucacagccugcuuggauuagaU..................   11      1     Fm2
.......................................................................aucacagccugcuuggauuaga...................   12      0     Fm2

....................................................................cauaucacagccugcuuggauuag....................   1       0     Mm2
.....................................................................auaucacagccugcuuggauua.....................   1       0     Mm2
.....................................................................auaucacagccugcuuggauuag....................   1       0     Mm2
......................................................................uaucacagccugcuugga........................   1       0     Mm2
......................................................................uaucacagccugcuuggaA.......................   2       1     Mm2
......................................................................uaucacagcUugcuuggau.......................   1       1     Mm2
......................................................................uaucacagccugcuuggau.......................   21      0     Mm2
......................................................................uaucacagccugcuuggUu.......................   1       1     Mm2
......................................................................uaucacagccugcuugUau.......................   3       1     Mm2
......................................................................uaucacagccugcuuggauu......................   170     0     Mm2
......................................................................uaucacagUcugcuuggauu......................   1       1     Mm2
......................................................................uaucacagcUugcuuggauu......................   1       1     Mm2
......................................................................uaucacagccugcuuggGuu......................   1       1     Mm2
......................................................................uaucacagccugcuuggauA......................   1       1     Mm2
......................................................................uaucacagccugcuCggauu......................   1       1     Mm2
......................................................................uaucacagccugUuuggauu......................   1       1     Mm2
......................................................................uaucacGgccugcuuggauu......................   2       1     Mm2
......................................................................uaucacagccugcuuggCuu......................   1       1     Mm2
......................................................................uaucacagccugcuuUgauu......................   1       1     Mm2
......................................................................uaucacagccugcuuggaAu......................   1       1     Mm2
......................................................................uaucacagccugcuuggauuG.....................   3       1     Mm2
......................................................................uaucacagccugcuuggauuU.....................   13      1     Mm2
......................................................................uaucacagccugcuuggauua.....................   131     0     Mm2
......................................................................uaucacGgccugcuuggauua.....................   4       1     Mm2
......................................................................uaucacagccugcuuggGuua.....................   1       1     Mm2
......................................................................uaucacagccAgcuuggauua.....................   1       1     Mm2
......................................................................uaucacagccugcuuggUuua.....................   1       1     Mm2
......................................................................uaucacagccugcuuggauuag....................   10824   0     Mm2
......................................................................uaucacagccugcuuggauuaA....................   8       1     Mm2
......................................................................uaucacagcUugcuuggauuag....................   10      1     Mm2
......................................................................uauAacagccugcuuggauuag....................   3       1     Mm2
......................................................................uaucacagccugcuuggauuaC....................   1       1     Mm2
......................................................................uaucacagccugcuuggauGag....................   1       1     Mm2
......................................................................uaucacagccuUcuuggauuag....................   3       1     Mm2
......................................................................uaucacagccugcCuggauuag....................   1       1     Mm2
......................................................................uaAcacagccugcuuggauuag....................   1       1     Mm2
......................................................................uaucacNgccugcuuggauuag....................   2       1     Mm2
......................................................................uaucGcagccugcuuggauuag....................   2       1     Mm2
......................................................................uaucacagccugcuuUgauuag....................   3       1     Mm2
......................................................................uaucacagccugcuugAauuag....................   27      1     Mm2
......................................................................uaucacagccugUuuggauuag....................   12      1     Mm2
......................................................................uUucacagccugcuuggauuag....................   1       1     Mm2
......................................................................uaucacagccugcuuggUuuag....................   42      1     Mm2
......................................................................uaucacagccugcuCggauuag....................   1       1     Mm2
......................................................................uauUacagccugcuuggauuag....................   2       1     Mm2
......................................................................uaucacagccugcuuAgauuag....................   2       1     Mm2
......................................................................uaucacagccugcGuggauuag....................   1       1     Mm2
......................................................................uaucacagccugcuGggauuag....................   2       1     Mm2
......................................................................uaGcacagccugcuuggauuag....................   3       1     Mm2
......................................................................uaucaUagccugcuuggauuag....................   5       1     Mm2
......................................................................uaucacagccugcuugUauuag....................   20      1     Mm2
......................................................................uaucacagccugcuugCauuag....................   40      1     Mm2
......................................................................uaucacagccCgcuuggauuag....................   11      1     Mm2
......................................................................uaucacagccGgcuuggauuag....................   20      1     Mm2
......................................................................Naucacagccugcuuggauuag....................   5       1     Mm2
......................................................................uaCcacagccugcuuggauuag....................   2       1     Mm2
```

```
                                  Star                                Mature
gagaaguguacaaugaaaguuuggcaucugccuucgauucaaguggcugugacguguugauuugguaucauaucacagccugcuuggauuagagguugguugucaguugugc
......................................................................uaucacagccugcuuggCuuag....................    50     1     Mm2
......................................................................uaucacagccugcuuggGuuag....................    28     1     Mm2
......................................................................uaucacagcAugcuuggauuag....................    1      1     Mm2
......................................................................uaucacagUcugcuuggauuag....................    41     1     Mm2
......................................................................uaucacagccugcuuggauuGg....................    7      1     Mm2
......................................................................uaucacagccuAcuuggauuag....................    1      1     Mm2
......................................................................uaucacagccugcuuggauCag....................    2      1     Mm2
......................................................................uaucacagccugcAuggauuag....................    3      1     Mm2
......................................................................Aaucacagccugcuuggauuag....................    2      1     Mm2
......................................................................uaucacagccugcuuggauuCg....................    2      1     Mm2
......................................................................uaucacagccugcuuggauuaU....................    11     1     Mm2
......................................................................uaucacagccAgcuuggauuag....................    21     1     Mm2
......................................................................uaucacagccugGuuggauuag....................    1      1     Mm2
......................................................................uCucacagccugcuuggauuag....................    1      1     Mm2
......................................................................uaucacaUccugcuuggauuag....................    3      1     Mm2
......................................................................uaucCcagccugcuuggauuag....................    1      1     Mm2
......................................................................uaucacagccugcuuggauuUg....................    2      1     Mm2
......................................................................uaucacGgccugcuuggauuag....................    33     1     Mm2
......................................................................Caucacagccugcuuggauuag....................    2      1     Mm2
......................................................................uauGacagccugcuuggauuag....................    2      1     Mm2
......................................................................uaucacagccugcuugUauuaga...................    2      1     Mm2
......................................................................uaucaNagccugcuuggauuaga...................    1      1     Mm2
......................................................................uaucacagccugcuuggauuGga...................    1      1     Mm2
......................................................................uaucacagccugcuuggGuuaga...................    9      1     Mm2
......................................................................uaucacagccugcuugCauuaga...................    12     1     Mm2
......................................................................Caucacagccugcuuggauuaga...................    2      1     Mm2
......................................................................uaucacagcUugcuuggauuaga...................    1      1     Mm2
......................................................................uaucacGgccugcuuggauuaga...................    9      1     Mm2
......................................................................uaucacagccugUuuggauuaga...................    5      1     Mm2
......................................................................uaucaUagccugcuuggauuaga...................    1      1     Mm2
......................................................................uaucacagccugcuuggUuuaga...................    10     1     Mm2
......................................................................uaucacagUcugcuuggauuaga...................    4      1     Mm2
......................................................................uauGacagccugcuuggauuaga...................    1      1     Mm2
......................................................................uaucacagccugcuugAauuaga...................    6      1     Mm2
......................................................................uaucacagccCgcuuggauuaga...................    5      1     Mm2
......................................................................uaucacagccugcuuggauuagU...................    448    1     Mm2
......................................................................uaucacNgccugcuuggauuaga...................    1      1     Mm2
......................................................................uaucacagccAgcuuggauuaga...................    6      1     Mm2
......................................................................uaucGcagccugcuuggauuaga...................    1      1     Mm2
......................................................................uaucacagccugcuuggCuuaga...................    13     1     Mm2
......................................................................uaucacagccugcuuggauuUga...................    1      1     Mm2
......................................................................uUucacagccugcuuggauuaga...................    1      1     Mm2
......................................................................uaucacagccugcuuggauuagC...................    1      1     Mm2
......................................................................uaucacagccugcuuggauuaga...................    2807   0     Mm2
......................................................................uaucacagccGgcuuggauuaga...................    1      1     Mm2
......................................................................uaucacagccugcuuggauuagG...................    16     1     Mm2
......................................................................uaucacagccugcuuggauuagag..................    1      0     Mm2
......................................................................uaucacagccugcuuggauuagaA..................    15     1     Mm2
......................................................................uaucacagccugcuuggauuagaU..................    9      1     Mm2
.......................................................................aucacagccGgcuuggauuag....................    1      1     Mm2
.......................................................................aucacagccugcuuggauuag....................    8      0     Mm2
.......................................................................aucacagccugcuuggauuaga...................    1      0     Mm2
.........................................................................Uacagccugcuuggauuag....................    1      1     Mm2

......................................................................uaucacagccugcuuAga........................    1      1     Mm1
......................................................................uaucacagccugcuuggau.......................    64     0     Mm1
......................................................................uauAacagccugcuuggau.......................    1      1     Mm1
......................................................................uaucacagccugcuuggGu.......................    1      1     Mm1
......................................................................uaucacagccAgcuuggau.......................    1      1     Mm1
......................................................................uaucacagccugcuugAau.......................    1      1     Mm1
......................................................................uaucacagccugcuuggauG......................    1      1     Mm1
......................................................................uaucacagccugcuuggCuu......................    2      1     Mm1
......................................................................uaucacagUcugcuuggauu......................    13     1     Mm1
......................................................................uaucacagccugcuuggGuu......................    1      1     Mm1
......................................................................uaucacagccugcuugCauu......................    1      1     Mm1
......................................................................uaucacagccugcuuggauu......................    430    0     Mm1
......................................................................uauAacagccugcuuggauu......................    1      1     Mm1
......................................................................uaucacagccCgcuuggauu......................    1      1     Mm1
......................................................................uaucacagccugcuuggauuU.....................    12     1     Mm1
......................................................................uaucacagccGgcuuggauua.....................    1      1     Mm1
```

```
                                  Star                                            Mature
gagaaguguacaaugaaaguuuggcaucugccuucgauucaaguggcugugacguguugauuugguaucauaucacagccugcuuggauuagagguugguugucaguugugc
......................................................................uaucacagccugcuuggCuua.....................  1     1  Mm1
......................................................................uaucacagccugcuuggauua.....................  80    0  Mm1
......................................................................Caucacagccugcuuggauuag....................  1     1  Mm1
......................................................................uaucUcagccugcuuggauuag....................  3     1  Mm1
......................................................................uaucacagccugcuuggauuUg....................  1     1  Mm1
......................................................................uaucacagccugcuuggauuaU....................  16    1  Mm1
......................................................................uaucacagccuUcuuggauuag....................  1     1  Mm1
......................................................................uaucacagccugcuuggCuuag....................  33    1  Mm1
......................................................................uaucacagUcugcuuggauuag....................  41    1  Mm1
......................................................................uaucacagccugUuuggauuag....................  1     1  Mm1
......................................................................uaucacagccugcuugUauuag....................  8     1  Mm1
......................................................................uaucacagccugcuugCauuag....................  12    1  Mm1
......................................................................uaucacagccugcuuUgauuag....................  1     1  Mm1
......................................................................uGucacagccugcuuggauuag....................  2     1  Mm1
......................................................................uaucacagccugcuuggauuGg....................  3     1  Mm1
......................................................................uUucacagccugcuuggauuag....................  1     1  Mm1
......................................................................uaucacaUccugcuuggauuag....................  2     1  Mm1
......................................................................uaucacagccCgcuuggauuag....................  4     1  Mm1
......................................................................uaucacagccugcuuggauuag....................  6010  0  Mm1
......................................................................uauAacagccugcuuggauuag....................  2     1  Mm1
......................................................................uaucaNagccugcuuggauuag....................  1     1  Mm1
......................................................................uaucacagccugcuugAauuag....................  15    1  Mm1
......................................................................uaucacGgccugcuuggauuag....................  18    1  Mm1
......................................................................Naucacagccugcuuggauuag....................  6     1  Mm1
......................................................................uaucacagcUugcuuggauuag....................  2     1  Mm1
......................................................................uaucacagccugcuuggauuaC....................  1     1  Mm1
......................................................................uaucacagccugcuuggGuuag....................  18    1  Mm1
......................................................................uaucacagccugcuuggaGuag....................  2     1  Mm1
......................................................................uaucacagccugcuuggaAuag....................  1     1  Mm1
......................................................................uaucaAagccugcuuggauuag....................  1     1  Mm1
......................................................................uaucacagccugcuuggUuuag....................  31    1  Mm1
......................................................................uaucacagccugcuAggauuag....................  1     1  Mm1
......................................................................uaCcacagccugcuuggauuag....................  1     1  Mm1
......................................................................uaucCcagccugcuuggauuag....................  1     1  Mm1
......................................................................uauUacagccugcuuggauuag....................  1     1  Mm1
......................................................................uCucacagccugcuuggauuag....................  1     1  Mm1
......................................................................uaAcacagccugcuuggauuag....................  3     1  Mm1
......................................................................uaucacagccugcuuggaCuag....................  1     1  Mm1
......................................................................uaucacagccGgcuuggauuag....................  3     1  Mm1
......................................................................uaucacagccAgcuuggauuag....................  6     1  Mm1
......................................................................uaucacagccugcuuggauGag....................  1     1  Mm1
......................................................................uaucacUgccugcuuggauuag....................  3     1  Mm1
......................................................................uaucacagccugcAuggauuag....................  1     1  Mm1
......................................................................uaucacagccugcuAggauuaga...................  1     1  Mm1
......................................................................uaucacagccugcuugAauuaga...................  3     1  Mm1
......................................................................uaucacagccugcuuggGuuaga...................  4     1  Mm1
......................................................................uaucaUagccugcuuggauuaga...................  1     1  Mm1
......................................................................uauAacagccugcuuggauuaga...................  1     1  Mm1
......................................................................uaucacagccugcuuggauuaga...................  1776  0  Mm1
......................................................................uaucacagccugcuuggauuagG...................  2     1  Mm1
......................................................................uaucacagccugcuuggCuuaga...................  5     1  Mm1
......................................................................uaucacagccugcAuggauuaga...................  1     1  Mm1
......................................................................uaucacagccugcuuggauuagU...................  750   1  Mm1
......................................................................uaucacagccGgcuuggauuaga...................  2     1  Mm1
......................................................................uaucacagccCgcuuggauuaga...................  1     1  Mm1
......................................................................uaucacagccugcuugUauuaga...................  3     1  Mm1
......................................................................uaucaGagccugcuuggauuaga...................  1     1  Mm1
......................................................................Naucacagccugcuuggauuaga...................  1     1  Mm1
......................................................................uaucacagUcugcuuggauuaga...................  16    1  Mm1
......................................................................Aaucacagccugcuuggauuaga...................  1     1  Mm1
......................................................................uaucacagccAgcuuggauuaga...................  7     1  Mm1
......................................................................uaucacagAcugcuuggauuaga...................  1     1  Mm1
......................................................................uaucacagccugcuuggUuuaga...................  6     1  Mm1
......................................................................uaucacagccugcuugCauuaga...................  5     1  Mm1
......................................................................uaucacagccugcuuggauuUga...................  1     1  Mm1
......................................................................uaucacagccugcuuggauuagaA..................  13    1  Mm1
......................................................................uaucacagccugcuuggauuagaU..................  10    1  Mm1
.......................................................................aucacagccugcuuggauuag....................  16    0  Mm1

..................................cgauucaaguggcuguga............................................................  1     0  Fm3
```

Star **Mature**

gagaaguguacaaugaaaguuuggcaucugccuucgauucaaguggcugugacguguugauuugguaucauaucacagccugcuuggauuagagguugguugucaguugugc			
..................................cgauuAaaguggcuguga..	1	1	Fm3
....auaucacagccugcuuggauuag....................	7	0	Fm3
....Cuaucacagccugcuuggauuag....................	6	1	Fm3
....uaucacagccugcuugga........................	5	0	Fm3
....uaucacagccugcuuggau.......................	379	0	Fm3
....uaucacagccugcuuggUu.......................	3	1	Fm3
....uaucacagccugcuugAau.......................	1	1	Fm3
....uaucacagccugcuuggaA.......................	9	1	Fm3
....uaucacagccGgcuuggau.......................	1	1	Fm3
....uaucacagUcugcuuggau.......................	6	1	Fm3
....uaucacagccugcuuggCu.......................	2	1	Fm3
....uaucacagccAgcuuggau.......................	1	1	Fm3
....uaucacagccugcuugCau.......................	1	1	Fm3
....uaucacagUcugcuuggauu......................	112	1	Fm3
....uaucacagcGugcuuggauu......................	1	1	Fm3
....uaucacagccAgcuuggauu......................	5	1	Fm3
....uaucacagccugcuuggGuu......................	2	1	Fm3
....uaucacagccGgcuuggauu......................	2	1	Fm3
....uaucacagccugcuuCgauu......................	1	1	Fm3
....uaucacagccugcuugUauu......................	3	1	Fm3
....uaucacagccugcuCggauu......................	1	1	Fm3
....uaucacagccugcuugCauu......................	5	1	Fm3
....Aaucacagccugcuuggauu......................	1	1	Fm3
....uaucacUgccugcuuggauu......................	1	1	Fm3
....uaucacagccugcuuggUuu......................	12	1	Fm3
....uaucacagccugcuuggCuu......................	15	1	Fm3
....uaucacCgccugcuuggauu......................	1	1	Fm3
....uaucacagccugcuuggauu......................	1851	0	Fm3
....uaucacagccugUuuggauu......................	8	1	Fm3
....uaucacagcUugcuuggauu......................	10	1	Fm3
....uaucacagccugcuuggauA......................	4	1	Fm3
....uaucacagccugcuuggaGu......................	2	1	Fm3
....uauAacagccugcuuggauu......................	1	1	Fm3
....uaucacagccCgcuuggauu......................	1	1	Fm3
....uaucacagccugcuuUgauu......................	5	1	Fm3
....uaucaUagccugcuuggauu......................	1	1	Fm3
....uaucacaUccugcuuggauu......................	1	1	Fm3
....uaGcacagccugcuuggauu......................	1	1	Fm3
....uGucacagccugcuuggauu......................	2	1	Fm3
....uaucacGgccugcuuggauu......................	3	1	Fm3
....Naucacagccugcuuggauu......................	1	1	Fm3
....uaucacagccugcuugAauu......................	4	1	Fm3
....uaucacagccCgcuuggauua.....................	1	1	Fm3
....uaucacagccugcuuggauua.....................	726	0	Fm3
....uaucacagccugcuuggCuua.....................	6	1	Fm3
....uaucacagUcugcuuggauua.....................	4	1	Fm3
....uaucacagAcugcuuggauua.....................	1	1	Fm3
....uaucacagccugcuuggauuU.....................	51	1	Fm3
....uaucacagccugcuuggauuG.....................	18	1	Fm3
....uaucacagccugcuugAauua.....................	3	1	Fm3
....uaucacagccugcuugCauua.....................	1	1	Fm3
....Caucacagccugcuuggauua.....................	1	1	Fm3
....uauAacagccugcuuggauua.....................	1	1	Fm3
....uaucacagccugcuuggauCa.....................	1	1	Fm3
....uaucacagccugcuuggGuua.....................	1	1	Fm3
....uaucacagccGgcuuggauua.....................	1	1	Fm3
....uaucacagcUugcuuggauua.....................	19	1	Fm3
....Aaucacagccugcuuggauuag....................	5	1	Fm3
....uaucacagccuCcuuggauuag....................	3	1	Fm3
....Caucacagccugcuuggauuag....................	12	1	Fm3
....uaucacagccCgcuuggauuag....................	82	1	Fm3
....uaucacagccugcuuggauuCg....................	2	1	Fm3
....uaucacagccugcuuUgauuag....................	22	1	Fm3
....uaucacagccGgcuuggauuag....................	121	1	Fm3
....uaucacagccugcuuggauuag....................	89373	0	Fm3
....uaucacagccugcuuggaCuag....................	9	1	Fm3
....uaucacagcAugcuuggauuag....................	11	1	Fm3
....uaucacagccugcGuggauuag....................	8	1	Fm3
....uUucacagccugcuuggauuag....................	14	1	Fm3
....uaucacagccugcuGggauuag....................	8	1	Fm3

```
                                   Star                                 Mature
gagaaguguacaaugaaaguuuggcaucugccuucgauucaaguggcugugacguguugauuugguaucauaucacagccugcuuggauuagagguugguugucaguugugc
......................................................................uaucacagccugcuuggauuGg....................   7     1   Fm3
......................................................................uaucacagccugcuuggauuUg....................   13    1   Fm3
......................................................................uaCcacagccugcuuggauuag....................   16    1   Fm3
......................................................................uaucacagccugcuuggauCag....................   11    1   Fm3
......................................................................uaucaNagccugcuuggauuag....................   14    1   Fm3
......................................................................uaucacagcGugcuuggauuag....................   4     1   Fm3
......................................................................uaucacagccugcuugNauuag....................   1     1   Fm3
......................................................................uaucacagccuUcuuggauuag....................   7     1   Fm3
......................................................................uaucacagccAgcuuggauuag....................   151   1   Fm3
......................................................................uauUacagccugcuuggauuag....................   27    1   Fm3
......................................................................uaucacagccugcuuggauuaU....................   84    1   Fm3
......................................................................uaGcacagccugcuuggauuag....................   18    1   Fm3
......................................................................uauAacagccugcuuggauuag....................   30    1   Fm3
......................................................................uaucacagccugcuuggauAag....................   2     1   Fm3
......................................................................uaucacagAcugcuuggauuag....................   5     1   Fm3
......................................................................uaucGcagccugcuuggauuag....................   11    1   Fm3
......................................................................uaucacagccugcuuggaAuag....................   5     1   Fm3
......................................................................uaucacagccuAcuuggauuag....................   20    1   Fm3
......................................................................uaAcacagccugcuuggauuag....................   18    1   Fm3
......................................................................uaucaAagccugcuuggauuag....................   1     1   Fm3
......................................................................uaucacagccugcCuggauuag....................   9     1   Fm3
......................................................................uaucacagccugcuugUauuag....................   145   1   Fm3
......................................................................uaucacagccugcuuggauGag....................   5     1   Fm3
......................................................................uaucacagccugcuuCgauuag....................   6     1   Fm3
......................................................................uaucacagccugcuuggCuuag....................   469   1   Fm3
......................................................................uCucacagccugcuuggauuag....................   7     1   Fm3
......................................................................Naucacagccugcuuggauuag....................   48    1   Fm3
......................................................................uNucacagccugcuuggauuag....................   1     1   Fm3
......................................................................uauGacagccugcuuggauuag....................   15    1   Fm3
......................................................................uaucacagccugcuugCauuag....................   237   1   Fm3
......................................................................uaucacagccugcuuggauuaC....................   4     1   Fm3
......................................................................uaucacaAccugcuuggauuag....................   10    1   Fm3
......................................................................uaucCcagccugcuuggauuag....................   18    1   Fm3
......................................................................uaucacNgccugcuuggauuag....................   3     1   Fm3
......................................................................uaucacagUcugcuuggauuag....................   554   1   Fm3
......................................................................uaucacagccugcuuggGuuag....................   269   1   Fm3
......................................................................uaucacagccugcuuggauuaA....................   7     1   Fm3
......................................................................uaucacagGcugcuuggauuag....................   5     1   Fm3
......................................................................uaucacaCccugcuuggauuag....................   6     1   Fm3
......................................................................uaucacagccugcuuggUuuag....................   386   1   Fm3
......................................................................uaucacagccugcuugAauuag....................   179   1   Fm3
......................................................................uaucUcagccugcuuggauuag....................   15    1   Fm3
......................................................................uaucacCgccugcuuggauuag....................   1     1   Fm3
......................................................................Gaucacagccugcuuggauuag....................   3     1   Fm3
......................................................................uaucacaUccugcuuggauuag....................   32    1   Fm3
......................................................................uaucacGgccugcuuggauuag....................   196   1   Fm3
......................................................................uaucacagccugcAuggauuag....................   18    1   Fm3
......................................................................uGucacagccugcuuggauuag....................   20    1   Fm3
......................................................................uaucacagccugcuCggauuag....................   10    1   Fm3
......................................................................uaucaGagccugcuuggauuag....................   6     1   Fm3
......................................................................uaucacagccugUuuggauuag....................   87    1   Fm3
......................................................................uaucacagccugcuuggaGuag....................   3     1   Fm3
......................................................................uaucacagccugcuuAgauuag....................   6     1   Fm3
......................................................................uaucacagccugGuuggauuag....................   4     1   Fm3
......................................................................uaucacagccugAuuggauuag....................   4     1   Fm3
......................................................................uaucacUgccugcuuggauuag....................   7     1   Fm3
......................................................................uaucacagcUugcuuggauuag....................   69    1   Fm3
......................................................................uaucacagccugcuAggauuag....................   7     1   Fm3
......................................................................uaucaUagccugcuuggauuag....................   27    1   Fm3
......................................................................uaucacagccugUuuggauuaga...................   3     1   Fm3
......................................................................uaucacagccugcCuggauuaga...................   3     1   Fm3
......................................................................uaucacagccugcuCggauuaga...................   1     1   Fm3
......................................................................uaucacagccugcuuggaAuaga...................   4     1   Fm3
......................................................................Caucacagccugcuuggauuaga...................   2     1   Fm3
......................................................................uaucacagccugcGuggauuaga...................   2     1   Fm3
......................................................................uaucaUagccugcuuggauuaga...................   6     1   Fm3
......................................................................uaucacagccugcAuggauuaga...................   4     1   Fm3
......................................................................uaucacagccugcuuggauuagU...................   2629  1   Fm3
......................................................................uaucUcagccugcuuggauuaga...................   2     1   Fm3
......................................................................uaucacaUccugcuuggauuaga...................   1     1   Fm3
```

Star Mature

```
gagaaguguacaaugaaaguuuggcaucugccuucgauucaaguggcugugacguguugauuugguaucauaucacagccugcuuggauuagagguugguugucaguugugc
......................................................................uaucaAagccugcuuggauuaga...................   1       1   Fm3
......................................................................uaucacagcUugcuuggauuaga...................   11      1   Fm3
......................................................................uaucacaAccugcuuggauuaga...................   1       1   Fm3
......................................................................uaucGcagccugcuuggauuaga...................   5       1   Fm3
......................................................................uaucacagccugcuuggGuuaga...................   37      1   Fm3
......................................................................uaucCcagccugcuuggauuaga...................   1       1   Fm3
......................................................................uaucacagccAgcuuggauuaga...................   22      1   Fm3
......................................................................uUucacagccugcuuggauuaga...................   1       1   Fm3
......................................................................uaucacNgccugcuuggauuaga...................   1       1   Fm3
......................................................................uaucacagccGgcuuggauuaga...................   9       1   Fm3
......................................................................uaucacagccugcuuggUuuaga...................   45      1   Fm3
......................................................................uauGacagccugcuuggauuaga...................   2       1   Fm3
......................................................................uaucacagccugcuuggauuagC...................   1       1   Fm3
......................................................................uauUacagccugcuuggauuaga...................   3       1   Fm3
......................................................................uaucacagccugcuuggauCaga...................   2       1   Fm3
......................................................................uaucacagccugcuuAgauuaga...................   4       1   Fm3
......................................................................uaucacGgccugcuuggauuaga...................   17      1   Fm3
......................................................................uaucacagGcugcuuggauuaga...................   2       1   Fm3
......................................................................uaucacagccuCcuuggauuaga...................   1       1   Fm3
......................................................................uaCcacagccugcuuggauuaga...................   1       1   Fm3
......................................................................uaucacagccugcuuUgauuaga...................   2       1   Fm3
......................................................................Naucacagccugcuuggauuaga...................   2       1   Fm3
......................................................................uaucacagccugcuuggauuaCa...................   1       1   Fm3
......................................................................uaucacagUcugcuuggauuaga...................   41      1   Fm3
......................................................................uaGcacagccugcuuggauuaga...................   4       1   Fm3
......................................................................uaucacagccugcuuggauuaUa...................   4       1   Fm3
......................................................................uaucacagccugcuuggauuUga...................   1       1   Fm3
......................................................................uaucacagccCgcuuggauuaga...................   8       1   Fm3
......................................................................uaucacagccugcuuggauuaga...................   10054   0   Fm3
......................................................................uaAcacagccugcuuggauuaga...................   2       1   Fm3
......................................................................uaucacagccugcuuggauGaga...................   1       1   Fm3
......................................................................Aaucacagccugcuuggauuaga...................   1       1   Fm3
......................................................................uaucacagccugcuugCauuaga...................   23      1   Fm3
......................................................................uaucacagccugcuugUauuaga...................   12      1   Fm3
......................................................................uaucacagccugcuugAauuaga...................   16      1   Fm3
......................................................................uauAacagccugcuuggauuaga...................   6       1   Fm3
......................................................................uaucacagccugcuuggCuuaga...................   35      1   Fm3
......................................................................uaucaNagccugcuuggauuaga...................   1       1   Fm3
......................................................................uCucacagccugcuuggauuaga...................   2       1   Fm3
......................................................................uaucacagccugcuuggaGuaga...................   1       1   Fm3
......................................................................uaucacagccuUcuuggauuaga...................   1       1   Fm3
......................................................................uaucacagccuAcuuggauuaga...................   1       1   Fm3
......................................................................uaucaGagccugcuuggauuaga...................   1       1   Fm3
......................................................................uaucacagcGugcuuggauuaga...................   1       1   Fm3
......................................................................uaucacagccugcuuggauuagaA..................   14      1   Fm3
......................................................................uaucacagccugcuuggauuagaU..................   4       1   Fm3
.......................................................................aucacagccugcuuggauua.....................   10      0   Fm3
.......................................................................aucacagccugcuuggaGuag....................   1       1   Fm3
.......................................................................aucacagccugcuCggauuag....................   1       1   Fm3
.......................................................................aucGcagccugcuuggauuag....................   7       1   Fm3
.......................................................................aucacagccugcuuggauuag....................   98      0   Fm3
.......................................................................aucacagccuCcuuggauuag....................   1       1   Fm3
.......................................................................aucacagccugcuuggauuagU...................   9       1   Fm3
........................................................................ucacagccugcuuggauu......................   2       0   Fm3
........................................................................ucacagccugcuuggauuag....................   6       0   Fm3
..........................................................................acagccugcuuggauuag....................   3       0   Fm3

.....................................................................auaucacagccugcuuggauuag....................   6       0   Mm3
.....................................................................Cuaucacagccugcuuggauuaga...................   1       1   Mm3
......................................................................uaucacagccugcuugga........................   2       0   Mm3
......................................................................uaucacagccugcuugCau.......................   1       1   Mm3
......................................................................uaucacagccugcuuggaA.......................   2       1   Mm3
......................................................................uaucacagccugcuuggau.......................   51      0   Mm3
......................................................................uaucacagccGgcuuggau.......................   1       1   Mm3
......................................................................uaucacagccugcuuggCu.......................   1       1   Mm3
......................................................................uaucacagcUugcuuggau.......................   5       1   Mm3
......................................................................uaucacagccugcuuggauG......................   1       1   Mm3
......................................................................uaucacagUcugcuuggauu......................   8       1   Mm3
......................................................................uaucacagccugcuuggCuu......................   4       1   Mm3
......................................................................uaucacagccugcuugUauu......................   1       1   Mm3
```

```
                                  Star                                 Mature
gagaaguguacaaugaaaguuuggcaucugccuucgauucaaguggcugugacguguugauuuugguaucauaucacagccugcuuggauuagagguugguugucaguugugc
.......................................................................uaucacagccugcuugAauu......................	1	1	Mm3
.......................................................................uaucacagccCgcuuggauu......................	1	1	Mm3
.......................................................................uaucacagccugcuuggGuu......................	1	1	Mm3
.......................................................................uaucacagccugcuuggUuu......................	1	1	Mm3
.......................................................................uaucacagccugcuuggauA......................	1	1	Mm3
.......................................................................uaucacagccugcuuggauu......................	304	0	Mm3
.......................................................................uaucacagccugcuugCauu......................	1	1	Mm3
.......................................................................uaucacagcUugcuuggauu......................	1	1	Mm3
.......................................................................uaucacagccugcuuUgauu......................	5	1	Mm3
.......................................................................uaucacagccugcuuggauua.....................	105	0	Mm3
.......................................................................uaucacagccugcuuggauuU.....................	16	1	Mm3
.......................................................................uaucacagccugcuuggauuG.....................	5	1	Mm3
.......................................................................uaucacagccugUuuggauua.....................	1	1	Mm3
.......................................................................uaucGcagccugcuuggauuag....................	1	1	Mm3
.......................................................................uaucCcagccugcuuggauuag....................	1	1	Mm3
.......................................................................uaucacagccugcuuggauuaU....................	13	1	Mm3
.......................................................................uaucacaAccugcuuggauuag....................	2	1	Mm3
.......................................................................uaucacagccugAuuggauuag....................	1	1	Mm3
.......................................................................uaucacCgccugcuuggauuag....................	3	1	Mm3
.......................................................................uaucacagccugcuuggCuuag....................	58	1	Mm3
.......................................................................uaucacagccugcuuggaGuag....................	1	1	Mm3
.......................................................................uaucacagccugcuuggauCag....................	2	1	Mm3
.......................................................................uaucacGgccugcuuggauuag....................	24	1	Mm3
.......................................................................uaucacagccugcuAggauuag....................	2	1	Mm3
.......................................................................uaCcacagccugcuuggauuag....................	3	1	Mm3
.......................................................................uaucacagccAgcuuggauuag....................	31	1	Mm3
.......................................................................uaucacagccGgcuuggauuag....................	12	1	Mm3
.......................................................................uaucacagccugcuuggUuuag....................	55	1	Mm3
.......................................................................uauUacagccugcuuggauuag....................	3	1	Mm3
.......................................................................uauGacagccugcuuggauuag....................	3	1	Mm3
.......................................................................uaucacagccugcuuggauuag....................	12309	0	Mm3
.......................................................................uaucacagAcugcuuggauuag....................	1	1	Mm3
.......................................................................uaucacagccugcuugUauuag....................	20	1	Mm3
.......................................................................uaucacagccugcAuggauuag....................	2	1	Mm3
.......................................................................uaucacaCccugcuuggauuag....................	1	1	Mm3
.......................................................................uaucacagccugcuuggauuUg....................	2	1	Mm3
.......................................................................uaucacagccugcCuggauuag....................	1	1	Mm3
.......................................................................Naucacagccugcuuggauuag....................	7	1	Mm3
.......................................................................uaAcacagccugcuuggauuag....................	5	1	Mm3
.......................................................................uauAacagccugcuuggauuag....................	5	1	Mm3
.......................................................................uaucaUagccugcuuggauuag....................	4	1	Mm3
.......................................................................uaGcacagccugcuuggauuag....................	1	1	Mm3
.......................................................................uaucacagccugcuuUgauuag....................	2	1	Mm3
.......................................................................uaucacagccugcuuggauuCg....................	1	1	Mm3
.......................................................................uaucacaUccugcuuggauuag....................	3	1	Mm3
.......................................................................uaucaNagccugcuuggauuag....................	3	1	Mm3
.......................................................................uaucacagcGugcuuggauuag....................	1	1	Mm3
.......................................................................uaucacagUcugcuuggauuag....................	55	1	Mm3
.......................................................................uaucacagccuUcuuggauuag....................	3	1	Mm3
.......................................................................uaucUcagccugcuuggauuag....................	2	1	Mm3
.......................................................................uaucacagccugUuuggauuag....................	12	1	Mm3
.......................................................................uaucacagccugcuuggauuaA....................	4	1	Mm3
.......................................................................uaucacagccugcuuCgauuag....................	1	1	Mm3
.......................................................................uaucacagccugcuuggauuGg....................	8	1	Mm3
.......................................................................uaucacagccugcuugCauuag....................	39	1	Mm3
.......................................................................uaucacagcUugcuuggauuag....................	7	1	Mm3
.......................................................................uaNcacagccugcuuggauuag....................	1	1	Mm3
.......................................................................uCucacagccugcuuggauuag....................	1	1	Mm3
.......................................................................uaucacagccCgcuuggauuag....................	19	1	Mm3
.......................................................................uaucacagccugcuugAauuag....................	24	1	Mm3
.......................................................................uaucacNgccugcuuggauuag....................	2	1	Mm3
.......................................................................uGucacagccugcuuggauuag....................	5	1	Mm3
.......................................................................uaucacagGcugcuuggauuag....................	1	1	Mm3
.......................................................................Caucacagccugcuuggauuag....................	3	1	Mm3
.......................................................................uaucacagccugcuuggGuuag....................	32	1	Mm3
.......................................................................uaucacagccugcuuggauuagC...................	4	1	Mm3
.......................................................................uaucacagccugcuCggauuaga...................	1	1	Mm3
.......................................................................uaucacagccCgcuuggauuaga...................	1	1	Mm3
.......................................................................uaucaNagccugcuuggauuaga...................	2	1	Mm3
.......................................................................uaucacagcUugcuuggauuaga...................	6	1	Mm3
```

Star **Mature**

```
gagaaguguacaaugaaaguuuggcaucugccuucgauucaaguggcugugacguguugauuugguaucauaucacagccugcuuggauuagagguugguugucaguugugc
......................................................................uaucacagccugcuuggaAuaga...................   1     1   Mm3
......................................................................uaucacagccugUuuggauuaga...................   3     1   Mm3
......................................................................uaucacagccugcAuggauuaga...................   1     1   Mm3
......................................................................uaucacagccGgcuuggauuaga...................   5     1   Mm3
......................................................................uaucacagccAgcuuggauuaga...................   6     1   Mm3
......................................................................uaucaUagccugcuuggauuaga...................   1     1   Mm3
......................................................................uaucacagccugcuuggauuaga...................   3281  0   Mm3
......................................................................uCucacagccugcuuggauuaga...................   1     1   Mm3
......................................................................uaucacagccugcuugUauuaga...................   6     1   Mm3
......................................................................uaucacagccugcuuggauuaUa...................   1     1   Mm3
......................................................................uaucacNgccugcuuggauuaga...................   1     1   Mm3
......................................................................uaucacagccugcuuggGuuaga...................   3     1   Mm3
......................................................................uaucacagccugcuuggaCuaga...................   1     1   Mm3
......................................................................uaucacagccugcuuUgauuaga...................   1     1   Mm3
......................................................................uaucacagccuUcuuggauuaga...................   2     1   Mm3
......................................................................uaucacagccugcuuggUuuaga...................   11    1   Mm3
......................................................................uaucacGgccugcuuggauuaga...................   6     1   Mm3
......................................................................uauGacagccugcuuggauuaga...................   1     1   Mm3
......................................................................uaucacagccugcuuggCuuaga...................   14    1   Mm3
......................................................................uaucacagccugcuuggauuagU...................   1029  1   Mm3
......................................................................uauAacagccugcuuggauuaga...................   2     1   Mm3
......................................................................uaucacagccugcuuggauuagG...................   14    1   Mm3
......................................................................uaucacagccugcuugCauuaga...................   13    1   Mm3
......................................................................uaucacagcAugcuuggauuaga...................   1     1   Mm3
......................................................................uaAcacagccugcuuggauuaga...................   1     1   Mm3
......................................................................uauUacagccugcuuggauuaga...................   3     1   Mm3
......................................................................uaucacagccugcuugAauuaga...................   7     1   Mm3
......................................................................uaucacagccugcuuggauAaga...................   1     1   Mm3
......................................................................uaucacagUcugcuuggauuaga...................   9     1   Mm3
......................................................................uaucacCgccugcuuggauuaga...................   1     1   Mm3
......................................................................uGucacagccugcuuggauuaga...................   1     1   Mm3
......................................................................uaucacagccugcuuggauuaAa...................   1     1   Mm3
......................................................................uaucacagccugcuuggauuagaA..................   3     1   Mm3
......................................................................uaucacagccugcuuggauuagaU..................   3     1   Mm3
......................................................................uaucacagccugcuuggauuagag..................   2     0   Mm3
......................................................................uaucacagccugcuuggauuagagU.................   1     1   Mm3
.......................................................................aucacagccugcuuggauu......................   1     0   Mm3
.......................................................................aucacagccugcuuggauuaU....................   1     1   Mm3
.......................................................................aucacagccugcuuggauuag....................   9     0   Mm3
.......................................................................aucacagccugcuuggUuuag....................   1     1   Mm3
........................................................................ucacagccugcuuggauu......................   1     0   Mm3
........................................................................ucacagccugcuuggauuaga...................   1     0   Mm3
........................................................................ucacagccugcuuggauuagagU.................   2     1   Mm3
..........................................................................acagccugcuuggauuaga...................   1     0   Mm3
```
